# The global psychological and physical effects of domestic abuse and violence on South Asian women: a qualitative systematic review

**DOI:** 10.3389/fgwh.2024.1365883

**Published:** 2024-07-09

**Authors:** Marina Masih, Christopher Wagstaff, Harjinder Kaur-Aujla

**Affiliations:** College of Medical and Dental Sciences, Institute of Clinical Sciences School of Nursing and Midwifery University of Birmingham Edgbaston, University of Birmingham, Birmingham, United Kingdom

**Keywords:** mental health, sikh, South Asian, physical injury, physical health and mental illness

## Abstract

**Introduction:**

The purpose of this review is to systematically extract and analyse global academic literature to determine the physical and psychological effects of domestic abuse and violence on South Asian women.

**Design:**

This review employs a qualitative systematic approach and thematic analysis to synthesize the narratives of affected women found in the literature. Given that domestic violence is often concealed and downplayed by various social factors, statistical reports and prevalence data offer only a limited view of the issue due to underreporting. Therefore, qualitative literature is deemed more dependable in this subjective domain as it captures and interprets the experiences and meanings within this under-studied group.

**Data sources:**

Embase, PsycINFO, Google Scholar, Web Of Science, MEDLINE, and ASSIA were searched.

**Review methods:**

All included studies were critically appraised using the CASP tool for qualitative research. Thematic analysis was conducted to develop six themes. Studies excluded did not address the research question, although intersecting population issues could be valuable topics for further research.

**Results:**

The literature indicates severe mental and physical health consequences of domestic violence and abuse, with some traits persisting long-term. The analysis underscores the significant role of resilience, suggesting that individuals can overcome traumatic social experiences without enduring lifelong labels or a deficit model.

**Conclusion:**

The findings provide support for future interventions aimed at recognizing signs of abuse and preventing severe psychological and physical consequences, particularly among South Asian women. Further research is needed to understand the impact on children and other family members affected by the victim's abuse, which falls beyond the scope of this review.

## 1 Introduction & background

Domestic violence and abuse encompass a range of coercive, threatening, controlling, degrading, and violent behaviours (1). Globally, intimate partner violence affects 641 million women and is the most common form of violence against women (2). In UK law and reports, the term is broadly recognized and includes various forms of abuse.

Demographically, the highest rates of Intimate Partner Violence (IPV) are recorded in South Asia, Sub-Saharan Africa, and Oceania, with prevalence rates between 33% and 51% among women aged 15–49 years (2). This study examines the impact of domestic violence on South Asian women, particularly in India, Pakistan, Bangladesh, Bhutan, Nepal, Afghanistan, the Maldives, and Sri Lanka (3). In South Asia, domestic violence remains a significant social issue, influenced by social, economic, and cultural factors that may normalize violence experiences (4). Research indicates that South Asian women are less likely to report violence due to the associated stigma, shame, fear of community isolation, and the importance placed on family reputation (5, 6). It is important to note that statistics do not capture unreported cases, making it challenging to assess the impact of these barriers on reporting behaviours. Therefore, this qualitative review will focus on the experiences of women who have participated in research, not those who remain undisclosed. The study also recognizes the distinct experiences of South Asian migrants and immigrants, acknowledging that both groups may have different experiences.

To summarize, the preliminary search for this review identified a single systematic review by Kalokhe et al. (7), which investigated the impact of domestic violence on physical and mental health. Focusing on 137 quantitative studies from India, it highlighted the need for a more extensive global search. The current review aims to compile and scrutinize all pertinent literature that describes the physical and psychological consequences of domestic abuse on South Asian women.

## 2 Methodology

### 2.1 Design

A qualitative systematic review of primary data was conducted to examine the physical and psychological effects of domestic violence and abuse on South Asian women. The aim was to comprehend the psychological and physical impacts through the lens of women's rich experiences. Consequently, a qualitative methodology was essential to foster understanding and provide insight into the researched situation, as the results are descriptive and interpretive (8). The review adhered to the “Preferred Reporting Items for Systematic Reviews and Meta-Analyses” (PRISMA) guidelines, which enhance the transparency, completeness, and accuracy of systematic review reporting (9). This review exclusively concentrated on qualitative data, analysing women's experiences via interviews, and included pertinent mixed-method studies. In this case, quantitative data were omitted, focusing solely on the analysis of interviews detailing lived experiences.

### 2.2 Search methods

The key themes of the research question were identified using the PEO framework. This includes identifying the population (P), exposure (E), and outcome (O) (10). The following research question was formulated…

1) What are the psychological and physical effects (O) of domestic violence and abuse (E) on South Asian women (P)?

The same search terms were utilized across all databases, but the format was adjusted to meet each database's specific requirements. The search terms, being relevant to the question were constructed using the themes that were identified utilising the PEO framework, (as seen in Table 1 below). Medical Subject Headings (MeSH) were employed in databases like MEDLINE, supplemented by keyword searches. Truncation was used to capture multiple suffix variations of a key term (8). To prevent bias in the search results and ensure no relevant literature was overlooked, searches were conducted without any pre-set limits (11).

**Table 1.** The PEO framework and the search terms that were used to answer the question.

Population	Exposure	Outcome
South-Asian women	Domestic Violence	Psychological effects
women	Abuse	Physical effects
females	Partner Violence	Mental health
Battered women	Physical abuse	Bodily effects
Pakistani	Spouse abuse	Psychological/physical impact
Indian	Family violence	Psychological trauma
Bangladeshi	Aggression	Outcomes (mesh)
Bhutanese	Intimate partner violence	Consequences (mesh)
Nepalese	Gender-based violence	Visible effects
Sri–Lankan		
Afghan women		
Maldivian women		

Beyond electronic database searches, additional strategies were employed to ensure a comprehensive literature review, following the recommendations of Cochrane (12) as well as Greenhalgh and Peacock (13). This included examining relevant journals at academic institutions and local libraries. A basic internet search with the search terms was also performed. Furthermore, the grey literature database OpenGrey (14) was explored using the keywords, but it yielded no pertinent literature, as noted by Bettany-Saltikov (10).

### 2.3 Screening

An online reference management website “Rayyan” was used to import the results of the screening searches. The exclusion and inclusion criteria (Table 2) were established before beginning the screening process. Once the results of the searches were uploaded, the titles and abstracts of the literature were examined, selecting those that met the predetermined inclusion criteria.

**Table 2.** A table showing the criteria established to assess the relevance of the literature and to maintain the focus of the review.

Inclusion Criteria	Exclusion Criteria
1) Primary Literature	Secondary Literature (reviews, books, newspaper articles, presentations etc)
2) Global literature	N/A
3) Literature published in English language	Literature not published or translated in English
4) South-Asian women	Men and children (0–17 years)
5) Qualitative data	Quantitative data
6) South-Asian women who have experienced or are experiencing any type of domestic abuse and violence	South-Asian women who have not experienced or are not experiencing any type of domestic abuse and violence
7) Literature focusing on the physical and psychological outcomes	Literature not focusing on the physical and psychological outcomes

### 2.4 Critical appraisal

The quality appraisal was based on criteria from the Critical Appraisal Skills Programme (CASP) tools for qualitative, cross-sectional studies, and case-controlled studies.

### 2.5 Data abstraction

Data extracted from each of the studies followed guidance by Cochrane (15). This included author(s), date, and location of publication, aim(s), design and data collection, sample, data analysis, key findings, and limitations (15). This is shown in Table 3.

**Table 3.** Table showing an analysis of the appropriate data extracted from each article.

Author(s), year, location of publication	Aim(s)	Design and data collection method	Sample	Data analysis	Key Findings	Limitations
Khan (2015) (16) Bangladesh	To explore the consequences of domestic violence against rural Bangladeshi women	Qualitative In-depth phenomenological interviews that lasted between 50-60 mins	39 female victims Snowball & purposive sampling	Thematic Analysis	• Domestic violence causes serious consequences to the overall health and wellbeing of individuals • Negative impact on the sexual, psychological, reproductive, physical, & behavioural health of women and can result in fatal consequences • Domestic violence also causes negative impacts to their children and family members	• Small sample size – not representative to the wider population • Used a male researcher to interview participants – the ease of disclosure of violence may have been affected due to the gender of the interviewer.
Hussain and Khan (2008) (17) Pakistan	Aim is not explicitly stated but within the abstract the research states it is exploring women's experiences of sexual violence in marital relationships and its outcomes on reproductive health	Qualitative • 1^st^ stage – interviewed key informants who were knowledgeable about community issues – other participants were identified • Unstructured interviews conducted with the key informants followed by focus group discussions with married women • Semi structured interviews with those women identified • Discussions were audiotaped and noted	24 female victims Snowball & random sampling Ages ranged from 20–70 years	Thematic Analysis	• Results showed non-consensual sex and sexual coercion was common • Unsafe abortions and unwanted pregnancies were seen as outcomes due to sexual violence, as well as excessive fatigue & abdominal pain • Women disclosed escalation of violence during pregnancy to be frequent.	• Small sample size – not representative to the wider population • No statement of ethical approval included • The use of random sampling was considered inappropriate due to the social sensitivities related with the topic & identifying and recruiting women who currently were experiencing sexual violence specifically. Thus, the chosen sample may not be representative of a larger population.
Chibber, Krishnan and Minkler (2011) (18) India	Aim is not explicitly described. However, within the body of the text, interview results of physicians' four core practices in response to Intimate partner violence (IPV) were displayed. This includes recognising indications of IPV such as physical injuries, psychological distress, vague symptoms, and sexual violence.	Qualitative Semi-structured in-depth interviews conducted which lasted appropriately 90 minutes at each physician's clinic. Discussions were transcribed and audiotaped	40 females Ages ranged from 28-30 years Purposive sampling	Grounded Theory Approach	• Common physical outcomes included cuts, bruises, burn marks, fractures, abdominal pain, headache, back ache, giddiness vomiting, insomnia, swollen or black eyes. • Psychological distress resulted in females being uneasy, withdrawn, fearful and unhappy. • Physicians' efforts could be improved with training that enhances counselling skills & their ability to link women with options of support.	• Small sample size – not representative to the wider population • Aim is not clearly stated making it harder to attain whether the research has answered the aim. • Physicians did not employ all 4 practices consistently, which included noticing indications of IPV & screening and assessments. Therefore, possibly making incorrect diagnoses or missing key information.
Ahmad-Stout et al. (2018) (19) United States	To describe the experiences linked with IPV among South Asian women in the United States.	Qualitative Semi-structured interview Interviews were recorded and transcribed	11 SouthAsian women Ages ranged from 24- 49 years Convenience sampling	Grounded Theory Approach	• Families and in-laws play an important role in the process of leaving the relationship – either support and/or pressure to remain in the relationship. • Facilitators to Leaving the Relationship included support from families and concerns for their children. • Long term effects of abuse included medical factors such as depression, suicidal ideation, loss of self-confidence, isolated from others, and withdrawal from daily living activities.	• Participants were recruited from domestic violence agencies. Results may differ from women who remain in abusive relationships or leave via nonformal means. • Small sample size
Kallivayalil (2010) (20) United States	Aim is not explicitly stated but within the abstract the research states the article examines the narratives from South-Asian immigrant survivors of domestic violence who accessed a mental health clinic.	Qualitative • Step 1 – in-depth interviews with 8 South-Asian immigrants • Step 2 – in-depth interviews conducted with 7 practitioners who worked with battered women	8 South-Asian women survivors and 7 practitioners who were South-Asian women Ages ranged from 30–40 years Snowball sampling	Grounded theory approach & narrative analysis	• Betrayal was an outstanding theme of suffering – betrayal of the faith women had placed in their husbands and in their in laws. • Intense rumination and rewinding of their marriage history & lives. • Self and other blame – women blamed themselves for the abuse, acquiring selfblame on their marriage. • Negative consequences on reproduction and mothering – forced abortions & unwanted pregnancies. • Psychological Distress Embodied in physical Symptoms – sleep difficulties, fainting, chronic chest pain, heart palpitations, extreme fatigue & gastric problems.	• Small sample size – not representative to the wider population • No statement of aim or ethical approval included • Possible inaccurate narratives – all participants had psychological therapy. Their understanding of experiences may have been developed to some degree by the therapeutic process. • It is likely that the length of time that women lived in the US. Recent immigrants or newly separated from their partners are perhaps less likely to have the opportunity to reflect on their history.
Najma and Naz (2018) (21) Pakistan	To examine how various types of domestic violence (sexual, physical, and psychological violence) impair the psychological well-being of the survivors. To recognise the causes of high prevalence rate of domestic violence in a Pakistani society.	Mixed method research design Stage 1: California Psychological Inventory's subscale of Well-being (Wb) (1) was used to assess the psychological wellbeing of women. Stage 2: Semistructured in-depth interviews conducted to analyse demographic factors in provoking domestic violence and psychological issues faced by women. Interviews were transcribed	100 survivor women Ages ranged from 50 years Purposive Convenience sampling	Quantitative Analysis: One-Way Analysis of Variance used Qualitative Analysis: Not stated as to how the in-depth interviews were analysed	Women had mostly impaired psychological well- being than physical, psychological, and economic violence as the graph showed low scores of physical and sexual violence. Demographic variables played a role in producing the prevalence rate of domestic violence which were social setup, disobeying, parental death, poverty, not having a son and illiteracy. Psychological problems faced included, low self-esteem, feelings of powerlessness, poor self-concept, sleep problems, helplessness, worthlessness, anhedonia, depression and posttraumatic stress disorder.	• Not clearly stated as to what approach was used to analyse qualitative data • Small sample size – not representative to the wider population
Hurwitz et al. (2006) (22) United States	The aim is not specifically written but the article states it wants to assess the associations between IPV and health outcomes of South-Asian women. As well as explore the nature of the health experiences from South-Asian women.	Mixed method research design Cross-sectional surveys with 208 women in relationships, assessed demographics, IPV history, and health. In-depth interviews were conducted with a separate sample of 23 women with a history of IPV, via telephone or face to face. Interviews were audiotaped and transcribed.	231 females all together Snowball & convenience sampling Women aged 18 or older	Quantitative Analysis: Assessed using logistic regression Qualitative Analysis: Grounded theory approach used	• Abused women were more likely than those with no history of IPV to report poor physical health. • Victimisation resulted in injury, depression, and chronic health concerns. This included anxiety affecting their appetite, sleep energy, and wellbeing.	• Generalisability limited by the use of a convenience sample – the sample (of 23) were women who had a history of IPV. • Women who were still with the abusive partner or had left their batterer and women who had not accessed formal services were not included. • Telephone interviews can result in inaccurate or potential loss of data. Some participants may present themselves in a socially desirable way.
Raj et al. (2010) (23) India	The aim is not specifically written but in the introduction the researcher states they want to examine experiences of perinatal abuse from in-laws and to assess associations between such experiences & IPV from husbands.	• Mixed method research design • Qualitative phase: face-to-face, semi-structured in-depth interviews with 32 women seeking health care for their infants (6 months or younger). • Quantitative phase: involved survey data collection from 1,038 mothers seeking immunisation for their infants • Interviews were transcribed & translated into English	• 1,070 women all together (But 32 women used for interviews) • No sampling method was stated. However, through the description given it is likely to be purposive sampling.	Quantitative Analysis: Assessed using Chi-square analyses & t-tests Qualitative Analysis: Grounded theory approach used	• 26.3% of the sample reported perinatal abuse (physical and nonphysical) from in-laws. • Non-physical forms of abuse included food denial, forced heavy domestic labour and prevention of medical care acquisition. • Physical health consequences included infant mortality, stillbirth, miscarriage, and low infant weight birth. • Perinatal abuse from in-laws is common amongst women in India and compromises the health of the child and maternal health.	Generalisability limited as only women who gave birth were included. Recruitment of women with infants aged 5–6 months provided inadequate time for full assessment for abuse during the post-partum stage. How common or severe abuse was after 56 months remains unknown. Findings do not directly associate with perinatal health consequences of abuse from in-laws. Other aspects of family violence including child abuse from women's natal family or spousal violence against the mother-in-law were not considered.

Following the process from Braun and Clark (24), an inductive approach was undertaken to analyse the data from the literature from where six themes were developed: visible injuries, reproductive health problems, temporary disabilities, psychosomatic symptoms, isolation, and withdrawal symptom and lastly, mental health disorders and suicidal ideation.

### 3.2 Critical appraisal of results

All studies included were appraised for quality, finding diversity in the quality of the articles. The results of the critical appraisal are displayed in Table 4. Out of eight studies, three explicitly stated receiving ethics committee approval or detailed the procedures to uphold ethical standards. Five studies did not mention ethical approval or participant confidentiality measures (17–20, 22). However, only Chibber et al. (18), Ahmad-Stout et al. (19), and Najma and Naz (21) clearly stated that they obtained consent. The publication dates of four studies (2006, 2008, 2010, and 2011) might reflect different publication guidelines from current standards, explaining the lack of explicit ethics statements. Additionally, the publication's location could influence the process of obtaining ethical approval, with low-income countries less likely to seek such approvals for health studies, as seen with Hussain and Khan (17) in Pakistan and Chibber et al. (18) in India (27). The need for ethical works for underserved communities is important and forefront of a good study.

**Table 4.** A table showing all the studies utilised that were appraised for quality.

Study Key: Y = yes N = No U = unclear	Was there a clear statement of the aims of the research?	Is a qualitative methodology appropriate?	Was the research design appropriate to address the aims of the research?	Was the recruitment strategy appropriate to the aims of the research?	Was the data collected in a way that addressed the research issue?	Has the relationship between researcher and participants been adequately considered?	Have ethical issues been taken into consideration?	Was the data analysis sufficiently rigorous?	Is there a clear statement of findings?	How valuable is the research?
Khan (16)	Y	Y	Y	Y	Y	N	Y	Y	Y	Research is valuable in highlighting the consequences of domestic violence from subjective experiences.
Hussain and Khan (17)	N	Y	Y	Y	Y	U	N	Y	Y	Research provides useful information into the link between violence and women's health and the impact of sexual violence on maternal and child health.
Chibber et al. (18)	N	Y	Y	Y	Y	Y	U	Y	Y	A valuable service evaluation in assessing the identification of IPV and the use of culturally appropriate practices to address it.
Ahmad-Stout et al. (19)	y	Y	Y	Y	Y	N	U	Y	Y	Provides useful insight into the IPV experiences and barriers faced in the help-seeking process.
Kallivayalil (20)	N	Y	Y	Y	Y	Y	N	Y	Y	Research is valuable in providing a beneficial perspective for understanding the context for violence in the South-Asian immigrant community.
Najma and Naz (21)	Y	Y	Y	Y	Y	U	Y	N	Y	Research is valuable in showing how physical and sexual violence has distressing effects on the psychological wellbeing of a victim.
Hurwitz et al. (22)	N	Y	Y	Y	Y	U	U	Y	Y	Research contributes to valuable findings on the injury and chronic health concerns caused by IPV.
Raj et al. (23)	N	Y	Y	Y	Y	Y	Y	Y	Y	Research is beneficial in showing how maternal and child health can be compromised by abuse from in-laws.

### 2.6 Data analysis

The six-step method by Braun and Clarke (24) gave the guidance for conducting a thematic analysis of the literature; this was selected due to its simplified and adapted method that helps in identifying themes within qualitative data (25). To reduce bias in the findings, an inductive technique was used to construct the themes from the literature based on the content of the data (24). This method recognised themes from the literature rather than predetermining them (26).

## 3 Findings

### 3.1 Summary of findings

**Figure 1:**
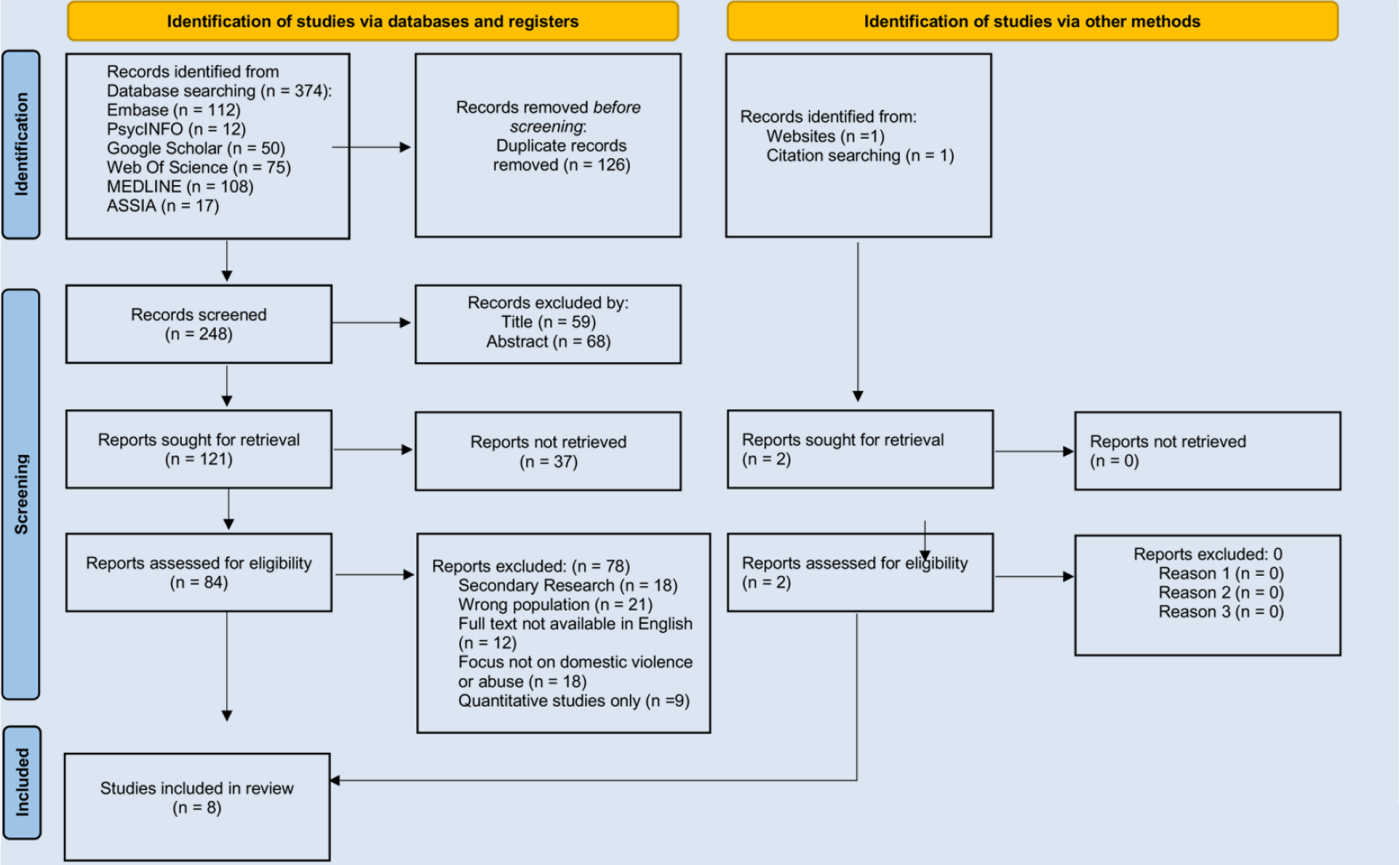
PRISMA 2020 flow diagram for new systematic reviews which included searches of databases, registers and other sources.

A total of 374 results were retrieved from six online databases, with an additional two results manually added, bringing the total to 376. The breakdown of results from the databases is as follows: Embase (112), PsycINFO (12), Web of Science (75), Google Scholar (50), MEDLINE (108), and ASSIA (17). Due to the large number of results from Google Scholar, only the first 50 were considered. The software “Rayyan” facilitated the exportation of results, during which 126 duplicates were removed. Further exclusions were made based on title (59) and abstract (68). After applying the predetermined inclusion and exclusion criteria, eight studies were selected for detailed review (Figure 1).

The included studies were conducted across four different countries from 2006 to 2018. Although this provides a wider range of information universally, generalisability is still limited as most of the data is present from countries of a South Asian background, where the highest prevalence rates of intimate partner violence are identified (2).

A common issue across all studies was the small sample size of women interviewed. This not only limits the generalizability of the results but also heightens the risk of type I or type II errors (28). It also undermines the validity of the results, as small sample sizes are unlikely to yield reliable outcomes (29). Nonetheless, within the context of this review, a small sample size may be considered suitable as it allows qualitative researchers to engage closely with participants, facilitating in-depth data collection through interviews (30).

Snowball sampling was utilized in four studies (16, 17, 20, 22), which heightens the risk of uncontrollable bias. This is because key workers or participants might recommend others who share their perspectives or experiences of abuse, potentially leading to a non-representative sample of the population (29). Additionally, purposive sampling was employed in three studies (16, 18, 21), which compromises study reliability and introduces significant bias, as researchers handpick participants that they deem most suitable (31).

Methodological issues were identified regarding the relationship between the researcher and participants not being considered. Interviewing female participants could have been affected using a male interviewer in the study conducted by Khan (16). This could result in the ease of disclosure of violence being affected (32). The risk of data being overlooked, or inaccurate information being reported, results in internal validity being reduced.

Only one study (22) conducted telephone interviews, which might have compromised the validity and reliability of the results due to the inability to observe the participants’ body language or facial expressions, potentially leading to data loss and distortion (29). This limitation could impair the interviewer's capacity to detect emotions such as discomfort, anger, or sorrow. Moreover, the difficulty in establishing rapport over the phone may also impact the quality of the findings (33). However, the fact that all interviews across the studies were recorded and transcribed enhanced inter-rater reliability, as it allows multiple researchers to participate in the analysis (34).

No studies were excluded because they were all deemed valuable and recognised as decent quality in helping to answer the research question.

### 3.3 Themes

#### 3.3.1 Physical

##### 3.3.1.1 Visible injuries

From the studies included in the review, one of the common themes was that women often described their experiences of physical violence as a use of force against their bodies resulting in visible outcomes. These ranged from minor to severe injuries. The discussion of visible injuries was seen in four studies (16, 18, 20, 22). Visible injuries varied from minor cuts and scratches (30) to more serious injuries such as swollen or black eyes, burn marks and bruises (16, 20, 22).

Severe head injuries which resulted in serious long-term consequences of neurological damage were reported. For example, one participant commented: “*he banged my head against a wooden pillar of the house. He did it several times. My skull hurt badly*” [(16), p.214]. Additionally, another visible long-term consequence was reported by a participant who stated her husband punched her in the face, knocking her front teeth out (16). Some injuries reported were acknowledged as not so distinct due to facial injuries not endured by the victim. For example, one participant stated “*he used to squeeze my thighs and leave marks*’’ [(20), p.804]. Another participant reported that her husband hit her with a stick several times, targeting her breasts and leaving scars (16).

##### 3.3.1.2 Reproductive health problems

A second theme that emerged from the interviews was that women suffered serious reproductive health problems resulting from domestic violence. Physical violence imposed by their partners was a dangerous experience for women during their pregnancy. The role of the mother-in-law in domestic violence is discussed in Kaur-Aujla (6), and evidence of extended family abuse is commonplace.

Miscarriages were associated with domestic violence in two studies (16, 23). A married 20-year-old participant discussed how her mother-in-law limiting her food intake during pregnancy resulted in a miscarriage: “*I was without food for two and half days; then I ran from my in-laws' place. I just went to my sister place. After a week I felt feverish and at that time my bleeding (miscarriage) started*” [(23), p.705]. Furthermore, another victim reported her husband kicking her abdomen and as a result, she experienced heavy bleeding and consequently suffered a miscarriage (16). Low infant birth weight was also identified and associated with reproductive health problems due to a victim's mother-in-law restricting her food intake (23).

Similarities were also noticed when victims reported unwanted forced pregnancies in three studies (16, 17, 20). One victim's husband did not like her using contraceptives and did not allow her to use any birth control methods: resulting in both her and her child suffering from chronic malnutrition (16). Hurwitz et al. (22) also documented malnutrition as a physical consequence. Hussain and Khan (17) found victims have difficulties in negotiating safe sex controlled by the husband which resulted in unwanted pregnancies, “*My husband still wants to have more children and wants to keep on having unprotected sex*” (p.474).

A widespread practice in dealing with unwanted pregnancies often led to the act of induced abortions as seen by Kallivayalil (16) and Chibber et al. (18). Evidentially, some victims had to terminate their pregnancies to contribute to the household income and continue working (18). Those that were unable to terminate their pregnancies gave birth to a stillborn baby. Even though the reason was unknown, she still blamed her husband; “*He used to do that thing (sex) during the whole period of my pregnancy. I gave birth to a stillborn baby. I am sure it is the consequence of his activity*” [(16), p.216].

##### 3.3.1.3 Temporary disabilities

Health consequences that kept victims incapacitated or out of work for a while were discussed across four studies (Table 5). Demonstratable outcomes of physical violence included longer-lasting medical effects such as requiring surgery due to broken bones (19). In another study, physician associates noticed indications of IPV through observations of broken limbs and fractures in their assessments (18). Fractures were also noted from one victim from Khan (16) who reported her husband fractured her leg after attacking her with a piece of stone. Similarly, another victim disclosed her husband fractured her leg after hitting her with an iron rod. During the interview, the researcher recognised that she could not walk normally (16). However, as this statement was from the researcher and not the victim, it questions how accurate and truthful this statement was, due to the presence of only one observer. Thus, lacking in inter-rater reliability (34). Mobility issues were reported by Hurwitz et al. (22); “*That day he hurt me too bad with the beer bottle, my ankle got hurt and I was not able to walk properly … There were times when I would go to class with the limping*” (p.255). This statement supports and shows how such profound consequences are affecting everyday life for participants and the resilience that victims show in continuing their daily activities despite coping with injury.

**Table 5.** A table showing how themes have been deduced and allocated through analysis.

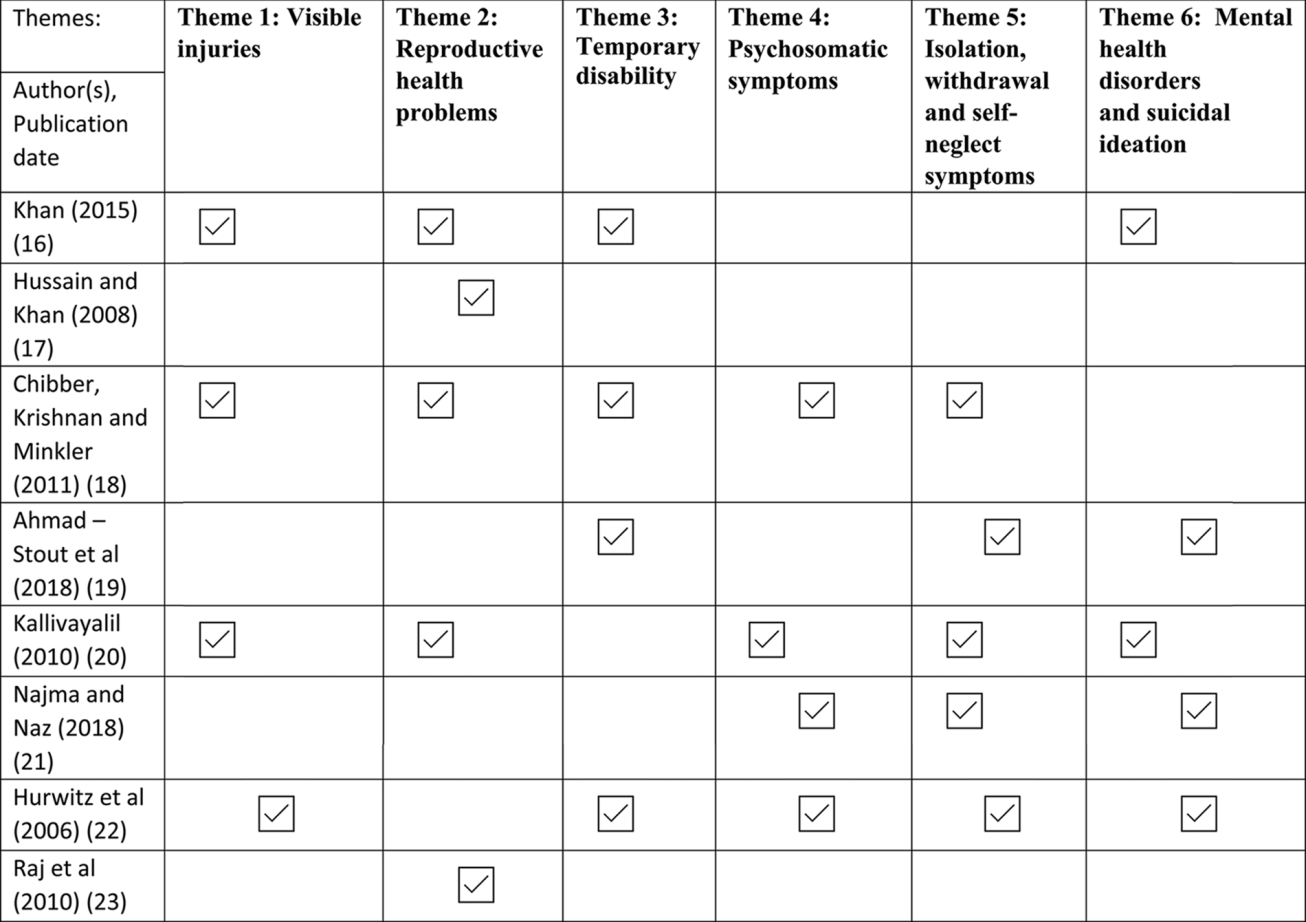

#### 3.3.2 Psychological

##### 3.3.2.1 Psychosomatic symptoms

Psychosomatic symptoms refer to physical health problems such as sleep disturbances, fatigue, respiratory problems, headaches, back pains, stomach aches, or unexplained pains. These do not have a physical cause but may have an emotional or mental cause (35).

Body aches in different forms varied between victims and in reporting mechanisms (18, 20, 22). Severe headaches, back aches and general body aches were frequently reported due to physical stress. Victims seamlessly associated their suffering narratives on both psychological and physical dimensions as they made meaning of their abuse experiences (20).

Difficulties in sleeping were frequently seen in the literature. In the sample, women who were no longer with their abusers explained how they are haunted by nightmares recalling the abuse and consequently fearing bedtime, “ *I still have the emotional stress. I mean, I even dream about the things that occurred with him when I was there… It is such a disturbed sleep now I do not want to go to bed*” [(22), p.257]. Similarly, another participant stated, “*I feel depressed, I cannot sleep*” [(20), p.803]. The findings from Najma and Naz (21) demonstrate the major psychological problems faced by women after experiencing domestic violence included sleep problems, however, the article did not state the type of qualitative analysis conducted and thus no elements of interviews were included, resulting in no understanding of contextual data.

Women often recognised their physical expression of stress and directly associated the stress they were experiencing with the breakdown of their bodies. For example, heart palpitations, gastric problems and respiratory problems identified as psychosomatic symptoms were described by a participant; “*Physically when you stress out too much, especially my body, I take everything inside me, I didn't share with no one. And finally, what happened, my stomach got upset and problems…. or I have a heart palpitation or something like my hands are blue, and I have breathing problem*” [(20), p.805]. This statement highlights how unexplained pains can relate to the mental well-being of the patient. Psychological distress was embodied in physical symptoms, and this is particularly important when assessing this group in medical situations.

##### 3.3.2.2 Isolation, withdrawal, and self-neglect symptoms

Isolation and withdrawal symptoms in the context of this theme are associated with symptoms of an already existing mental health condition, such as anxiety or depression. Individuals that are experiencing withdrawal or isolation will not participate in activities, they would usually enjoy being alone or with others (36). Indicators of isolation and withdrawal may include a change in appetite, lowered self-esteem, feeling helpless, lowered energy or motivation, recurring feelings of guilt, worthlessness, and self-loathing (37).

The loss of self-confidence was identified in two studies. Ahmad-Stout et al. (28) describe how a participant sample mentioned struggling with a reduced sense of confidence which they then associated with a feeling that they had lost a part of themselves. Female speakers explained how their lost capacity to enjoy life contributed to their impaired functioning: “(After the abuse) *I started to lose my self-confidence and the, because earlier I used to be very bubbly, full of energy. But then I … I just lost everything* …” [(22), p.257]. This statement also implies feelings of lowered energy and motivation.

Many victims also expressed feeling hopeless, helpless, and isolated from others even including their families of origin. “*I was feeling … helpless and just like my life doesn't make sense*” [(19), p.948]. Similarly, Najma and Naz (21) also found feelings of helplessness, worthlessness, and hopelessness to be emotions expressed by surviving women. Additionally, women who remained in the relationships were seen to display self-loathing behaviours; “*At first* (in the abusive relationship), *I questioned my own sanity and wondered if there was something wrong with me. I felt very helpless*’’ [(22), p.256]. However, these behaviours may have been induced because of the abuse and the perpetrator's gradual degradation of the victim.

Appetite and eating concerns were a recurring theme in the interviews shown in three studies (18, 20, 22). Isolation from family members often resulted in a change in eating behaviours as one woman described how she would cook for her children but not eat with them and preferred to sit in her room alone (20). Here, women described how the stress of abuse affected their appetite; “ *I lost my appetite* (in the abusive relationship). *I mean I was not able to eat properly. I mean because the emotional stress maybe*” [(22), p.257]. Similarly, loss of appetite was also reported by Chibber et al. (18).

Signs of psychological distress among women were noted as being uneasy, withdrawn, fearful or unhappy (18). Whereas Najma and Naz (21) highlighted key psychological issues faced by the survivors were anhedonia, poor self-concept, and low self-esteem, all of which were deemed appropriate to be categorised in this theme.

##### 3.3.2.3 Mental health disorders and suicidal ideation

A range of mental health disorders stated by victims were commonly reported through the interviews. Examples of reported diagnoses seen throughout the literature included depression and post-traumatic stress disorder (PTSD) can be with or without suicidal ideation or attempts. Three studies investigated PTSD because of domestic abuse and violence (20–22). Women described mental health concerns once they had left the relationship, “ *I have PTSD* (posttraumatic stress disorder) … *I've been losing some of my memory and I cannot pay attention to anything*…” [(22), p.256]. Women in this sample had episodes of PTSD and found it challenging to fulfil their roles as mothers (20). The findings from Najma and Naz (21) also found PTSD to be a significant psychological problem faced by survivor women. However, again, the pathologisation of women in these circumstances must be acknowledged and rather, trauma informed responses are key. In that, there are women who have been great mothers to their children despite incurring abuse, battling with mental health, and these successes are less emphasised in medical works due to the “disease model”, Syme and Hagen (38).

The diagnosis of depression was stated in four studies. IPV was significantly associated with mental health resulting in impacting the general health of individuals, “*My doctor says I am in depression. It does affect my health*” [(22), p.256]. Depression was stated by Ahmad-Stout et al. (19) and Najma and Naz (21) through suicidal attempts and thoughts. One woman also discussed how her depression because of her abortion caused her to neglect her family, “*I feel depressed, I cannot sleep, and I was negligent of the second child*” [(20), p.803].

Three studies showed how women often discussed suicide attempts as they thought death appeared to be a better option than life; “*one time I went to the river to commit suicide*” [(22), p.256]. Another participant described, “*I had a knife in my bathroom to slit myself*” (19). Ideas of taking their own life remained in women's thoughts, “*Often, I think so many people are dying every day so why not me*?” [(16), p.216].

## 4 Discussion

This qualitative systematic review sought to gather and analyse relevant studies on the impact of domestic abuse and violence against South Asian women (39). Although the quality of research varied, consistent themes emerged across the studies. These included visible injuries, reproductive health issues, temporary disabilities, psychosomatic symptoms, isolation, withdrawal symptoms, and mental health disorders with suicidal ideation. Nevertheless, Kaur-Aujla et al. (6) recommend exercising caution when applying mental health labels and pathologizing women who are coping with challenging life situations.

Upon reviewing the methodologies of the studies examined, it was found that the studies relied on interviews to gather data. The validity of these studies has been challenged due to the reliance on self-reported data, which subjects the qualitative findings to recall bias and an increase in social desirability bias (40). There is a possibility that participants may have responded in a manner they believed the researcher desired, thus not providing an accurate representation of their true opinions and thoughts. However, social desirability is more likely to lead to the underreporting rather than overreporting of domestic violence (41). It is advisable for researchers to incorporate a social desirability scale in the development of their interviews to reduce the tendency of socially desirable responses, thereby controlling for social desirability bias in data analysis (42). Furthermore, as previously indicated in the introduction, evidence suggests that women from South Asian backgrounds are less inclined to report violence due to the associated stigma and shame, as well as the importance placed on family reputation (6, 43). This reluctance to disclose may also contribute to the questioned validity, as nondisclosure can lead to a lack of belief or validation, causing additional trauma and re-traumatization.

This review confirms that psychological repercussions of domestic abuse, such as mental health problems and psychosomatic symptoms, are important health consequences. However, cultural conflict in South Asian women is frequently blamed for the emergence of these effects (6, 44). The validity of reporting somatisation symptoms, as observed in interviews with South Asian women, is debated since somatisation is a prevalent concept among non-Western populations (45). Kirmayer and Young (46) suggest that enhanced psychological insight could lead to fewer reported somatic symptoms. Women in these interviews frequently associated their psychological distress with physical ailments. Burr (47) recommends that such interpretations be examined to address both symptom sets thoroughly. It is posited that a lack of mental health literacy exists in South Asian communities, prompting the need for medical practitioners to screen all women for domestic violence and abuse. Nonetheless, the analysis of various studies indicates that psychological symptoms, particularly depression and anxiety, were consistently reported.

Ali et al. (48) observed that depression in married women was linked to marital rape, domestic abuse by in-laws, early marriage, and lack of autonomy in marriage decisions. Marital rape, as noted in this review, is associated with reproductive health issues. In South Asian countries like Bangladesh, Maldives, India, and Sri Lanka, there are no laws criminalizing marital rape (49), indicating that women are unable to report their husbands for such acts due to the absence of legal provisions. This relates to the earlier point that intimate partner violence is the most common form of violence against women, with South Asian women experiencing the highest rates of such violence. The systematic review's primary findings corroborate these observations.

It was found that physical health concerns stem both directly from injury and indirectly from the stress of domestic violence. Chronic problems included backache, headache, and other body pain such as gastrointestinal problems. Women stated these issues started during the abuse in the relationship and continued even when they were no longer with their partner. Other researchers support this finding that physical health problems persist long-term, even after the abuse has ended (33, 50, 51). Even though these physical injuries reported were common amongst all studies, it relates to the notion as discussed above, whether these symptoms such as headaches were a result of somatisation. Questions asked to participants should be discussed in advance, so physical and psychological symptoms are addressed comprehensively.

The findings from this review have demonstrated substantial severe consequences on women's health. Sadly, society has contributed to escalating the incident rates of domestic violence through old traditions and norms and propaganda. These findings are in line with Grose and Grabe (52) findings, which showed that adopting a nonserious attitude toward domestic violence allowed society to enhance its severity. This connects to the introduction's discussion of the persistence of domestic violence and how societal and cultural factors can normalise violent experiences (4). Thus, re-iterating the idea that this nonserious attitude toward domestic violence within the South Asian community, viewing domestic violence as normal in a key factor in the continuation of the issue.

Factors such as poverty and autonomy among women have been seen to provoke the occurrence rate of domestic violence. One study found that the failure to fulfil the basic needs of a man towards his family creates tension and stress which is released through acts of violence against his family (53). These findings are also consistent with findings from Purvin (54), and Keenan et al. (55) who found that women in low-income families or marriages, who experienced domestic violence continuously stayed in that abusive relationships, just because of their dependency on their husbands in terms of money. Sarkar (56) discovered that a husband's final say on household autonomy was a risk factor. A wife's autonomy in managing her earnings and having joint autonomy in household decision-making were protective factors. Therefore, among the males who had grown up with parental abuse, perceptions of relationship rights and autonomy in this study suggested a belief system that enabled the justification for “beating” their wives (56). As mentioned in the introduction, economic factors were found to be a partial result of domestic violence being a persistent issue and thus as a result leads to South Asian women finding it difficult to leave their marriage *in situ*ations, albeit this is not a heterogenous group and more specific intergroup work in needed.

Suicidal ideation and attempts were identified as a common recurring theme throughout the literature. It was identified that married South Asian women were specifically more at risk of suicide attempts due to isolation from others, gender stereotyping, cultural conflict, and poor self-esteem. Consequently, a clear and positive correlation between domestic abuse and mental health can be identified (57). However, as congruent as the evidence is, the pathologisation of women because of Western diagnostic criteria is fraught with frustration (6). Therefore further research is required to explore the sociocultural needs of this homogenous group given the global geopolitical diaspora.

## 5 Strengths and limitations

This review's strength lies in its comprehensive search, which utilized various databases, extensive internet searches, and hand-searching of reference lists, thus identifying a broad spectrum of literature. A limitation of this review is the possibility of bias introduced during the initial stages by a novice researcher, although this was later checked by a more experienced researcher. To reduce bias, strategies like using an inductive approach for developing themes, instead of a pre-established one, were employed. Additionally, the language proficiency of the author led to the exclusion of non-English papers due to limited resources and lack of translations. This resulted in the omission of potentially relevant articles for South Asian women in languages such as Punjabi, Hindi, and Bengali.

This review is exclusively cantered on qualitative data. This approach is necessary because quantitative measures might not accurately capture the experiences of victims, although they can help researchers identify actions that contribute to understanding the various forms of violence (58). Quantitative methods for investigating domestic violence often present challenges and lack depth, as they are tied to contextual factors like cultural beliefs and practices (59).

## 6 Conclusion

This review was conducted to examine the psychological and physical impacts of domestic abuse and violence on South Asian women. It involved a systematic search, critical appraisal, and thematic analysis of studies relevant to the objective. The study is both crucial and timely, as domestic violence has been recognized as a rising global issue, particularly after the COVID-19 pandemic, which saw an increase in cases during lockdown periods.

In summary, six themes emerged: visible injuries, reproductive health issues, temporary disabilities, psychosomatic symptoms, isolation, and withdrawal symptoms, and finally, mental health disorders and suicidal thoughts. These themes contribute to understanding the experiences of these women, which can guide future interventions aimed at recognizing abuse and, hopefully, preventing its severe consequences.

## Data Availability

The original contributions presented in the study are included in the article/Supplementary Material, further inquiries can be directed to the corresponding author.
